# Mechanical and Physicochemical Characteristics of a Novel Premixed Calcium Silicate Sealer

**DOI:** 10.3390/ma17133374

**Published:** 2024-07-08

**Authors:** Naji Kharouf, Filippo Cardinali, Raya Al-Rayesse, Ammar Eid, Ziad Moujaes, Mathilda Nafash, Hamdi Jmal, Frédéric Addiego, Youssef Haikel

**Affiliations:** 1 Department of Biomaterials and Bioengineering, INSERM UMR_S, Strasbourg University, 67000 Strasbourg, France; youssef.haikel@unistra.fr; 2 Department of Endodontics, Faculty of Dental Medicine, Strasbourg University, 67000 Strasbourg, France; 3 Private Practice, 60123 Ancona, Italy; filocardinali@gmail.com; 4 Department of Endodontics, Faculty of Dental Medicine, Damascus University, Damascus 0100, Syrian Arab Republic; raya.alrayes@damascusuniversity.edu.sy; 5 Department of Endodontics and Operative Dentistry, Faculty of Dentistry, International University for Science and Technology (IUST), Damascus 011, Syrian Arab Republic; ammarendo89@gmail.com; 6 Department of Endodontics, Beirut Arab University, Beirut 11-5020, Lebanon; ziadmoujaes@gmail.com; 7 Private Practice, San Dimas, CA 91773, USA; matildanafash@gmail.com; 8 ICube Laboratory, UMR 7357 CNRS, Mechanics Department, University of Strasbourg, 67000 Strasbourg, France; jmal@unistra.fr; 9 Luxembourg Institute of Science and Technology (LIST), Department Materials Research and Technology (MRT), ZAE Robert Steichen, 5 rue Bommel, L-4940 Luxembourg, Luxembourg; frederic.addiego@list.lu; 10 Pôle de Médecine et Chirurgie Bucco-Dentaire, Hôpital Civil, Hôpitaux Universitaire de Strasbourg, 67000 Strasbourg, France

**Keywords:** calcium silicate-based sealer, physicochemical properties, compressive strength, porosity, endodontic materials

## Abstract

The aim of the present in vitro study was to evaluate specific mechanical and physicochemical properties of three calcium silicate-based sealers, BioRoot™ Flow (BRF), CeraSeal (CRS) and TotalFill^®^ (TF). Samples were prepared to evaluate different physicochemical and mechanical properties of the tested sealers. These evaluations were accomplished by investigating the pH changes over time, porosity, roughness, flow properties, compressive strength and wettability. The results were statistically evaluated using one-way analysis of variance. All three sealers demonstrated an alkaline pH from 1 h of immersion in water to 168 h. A higher porosity and hydrophily were detected in BRF samples compared to CRS and TF. No significant difference was found between the tested materials in the flow properties. Lower compressive strength values were observed for BRF compared to TF and CRS. Differently shaped structures were detected on the three materials after 7 days of immersion in PBS. The three materials demonstrated a higher solubility than 3% after 24 h of immersion in water (CRS < BRF < TF). The novel premixed calcium silicate sealer (BRF) had comparable physicochemical properties to the existing sealers. The lower compressive strength values could facilitate the removal of these materials during retreatment procedures. Further studies should investigate the biological effects of the novel sealer.

## 1. Introduction

An optimal 3D obturation for a root canal is an essential parameter to achieve a successful endodontic treatment [1]. Suitable materials are used to obturate the root canal anatomy and seal against bacterial contamination, thus preventing the recontamination of the root canal system [2]. Different chemical compositions are used to fabricate the endodontic materials such as epoxy resin, zinc oxide-eugenol, calcium silicate and flowable gutta-percha [3]. These materials differ in terms of biological, setting and physicochemical reactions [1,2,3].

Calcium silicate (CS)-based materials, colloquially termed bioceramics, are widely used in endodontic treatments (by approx. 51.7% of dentists) [4]. Among these CS dental materials, mineral trioxide aggregate was the first CS cement, which was introduced in 1993 [5]. This CS form, called “Putty”, had a large particle size (typically in the range of 1.5–160 µm), engendering a poor flowability that was detrimental to dental sealer application [6]. The decrease in the particle sizes of these CS materials by using nanotechnology permitted a new generation of CS materials to be fabricated, which was introduced recently in the dental market [3,7]. Two forms of CS sealers are used: manually mixing sealers (powder–liquid) and ready-to-use sealers (premixed). However, several studies have identified problems related to manual mixing, such as alteration in the physicochemical properties of these materials [8]. Cavenago et al. [9] and Torres et al. [10] reported that a change in the ratio of powder–liquid could increase the solubility and porosity of these sealers and could impact the pH, calcium ion release, radiopacity and setting time. Accordingly, in 2008, designed to avoid errors in the mixing procedure, premixed ready-to-inject CS sealers were introduced in endodontic treatments. These premixed CS materials in both sealer [2,3] and putty [11,12] forms have since been widely used for their good biological reactions and biocompatibility.

BioRoot™ Flow (SEPTODONT Inc., Lancaster, PA, USA), which is commercialized in the USA, is the new version of the powder–liquid BioRoot™ RCS, which was extensively investigated in the past [13]. This novel premixed bioceramic is a permanent endodontic sealer that is biocompatible and ensures bioactive properties, an alkaline pH, crystallization and easy removal from the canal in retreatment process [13]. This new CS from SEPTODONT (Inc., Lancaster, PA, USA) was introduced as an alternative to the old powder–liquid version, and there is no study in the literature that yet evaluates this new material, including its physicochemical and mechanical properties.

CeraSeal (Meta Biomed Europe GmbH, Mülheim an der Ruhr, Germany) is also a premixed CS sealer that provides good biological properties, an appropriate filling ability and better physicochemical properties and homogeneity than powder–liquid CS sealers [2].

TotalFill^®^ (FKG Dentaire SA, La Chaux-de-Fonds, France), a premixed CS sealer, was the first CS sealer introduced in the European dental market [14]. This sealer has demonstrated appropriate biological and physicochemical properties in endodontic treatments.

All these materials, which will be in contact in the long term with dental tissues and oral cavity elements, should have an optimal biocompatibility, that is, the ability of the product to function in the oral cavity without causing harm. To achieve this, the host and the biomaterial must interact harmoniously, which is crucial for providing successful treatments and ensuring patient safety [15].

Against that background, the aim of this study was to evaluate the compressive strengths and physicochemical properties of three calcium silicate sealers. The null hypothesis was that there would be no difference between the three premixed calcium silicate-based sealers in terms of physicochemical and mechanical properties.

## 2. Materials and Methods

### 2.1. Materials

Three premixed calcium silicate-based sealers, BioRoot™ Flow (SEPTODONT Inc., Lancaster, PA, USA), CeraSeal (Meta Biomed Europe GmbH, Mülheim an der Ruhr, Germany) and TotalFill^®^ (FKG Dentaire SA, La Chaux-de-Fonds, France), were used in the present in vitro study (Table 1). This study was conducted in accordance with the Declaration of Helsinki.

### 2.2. Specimen Preparations

**Figure 1:**
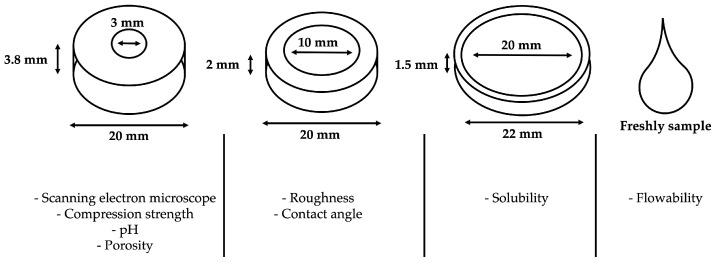
Graphical image demonstrates the various Teflon molds used for porosity, solubility, pH, compressive strength, scanning electron microscope, roughness, flow and contact angle analyses.

Different specimen dimensions were prepared by using various Teflon mold sizes (height/diameter: 3.8/3 mm, 2/10 mm and 2/20 mm) to evaluate the physicochemical and mechanical properties of the sealers used (Figure 1). Freshly prepared samples were used for the flow testing. Every sealer was injected into the different molds using its injection tip with glass slides underneath. All filled molds were kept in the dark for 72 h at 37 °C and humidity to allow for proper setting; then, different analyses were performed.

### 2.3. Evaluation of pH

Six specimens from each group were placed in 10 mL of distilled water. The samples were kept at 37 °C for 72 h. A pH meter (CyberScan pH 510, Thermo Scientific, Waltham, MA, USA) was used to record the pH of the water at 1, 24, 72 and 168 h. The pH meter was calibrated using standard solutions at pH 10, 4 and 7 (Hanna Instruments, Lingolsheim, France) before each set of measurements. The pH electrode was rinsed with distilled water to eliminate the previous solution.

### 2.4. Roughness

A digital profilometer (Keyence, Osaka, Japan) at 2500× magnification was used to investigate the roughness of each surface. The average roughness (Sa) was measured using the 7000 VHX software (Keyence, Osaka, Japan).

### 2.5. Wettability

Three specimens from each group were kept in a dry condition overnight. The samples were then used for the investigation of the sorption time, using a 5 µL drop of distilled water on the sealer surface, with a contact angle device (Biolin Scientific, Espoo, Finland). The profile of the water drop was recorded by a horizontal camera.

### 2.6. Scanning Electron Microscope (SEM) Analysis

Three samples from each group were placed in 10 mL of phosphate-buffered saline (PBS10x, Dominique Dutscher, Bernolsheim, France) for 7 days at 37 °C. After the period of immersion, the samples were washed gently for 5 min. All specimens were sputter-coated with gold–palladium using a Hummer JR sputtering device (Technics, San Jose, CA, USA). The specimens were observed at magnifications of x2000 and x8000 to evaluate the morphological changes in the sealer surface using a scanning electron microscope (SEM; Quanta 250 FEG, FEI Company, Eindhoven, The Netherlands”; 10 kV acceleration voltage of the electrons).

### 2.7. Solubility

Three samples of each group, following the standard ISO 6876:2012 [16], were weighed using a digital system (accuracy ± 0.0001 g) before a 24 h immersion period in 50 mL of distilled water at 37 °C. After 24 h, the samples were dried at 110 °C and weighed again to obtain the final weights. The solubility percentages were measured from the differences in mass between the final and the initial weights.

### 2.8. Flowability

Three samples for each group were used to determine the flowability of each sealer. The procedure was performed following ISO 6876/2012 [16], with 50 µL of each material dispensed on a glass plate (40 mm × 40 mm × 5 mm, 20 g). Then, a second similar glass plate was carefully placed on top of the materials, along with a weight of 100 g placed centrally on top of the second glass plate. After 10 min, the minimum and maximum diameters of the material between the two glass plates were measured using a digital caliper (Dexter, Elkhart, IN, USA). The mean diameter was calculated.

### 2.9. Porosity

The internal structures of the tested materials were investigated in 3D by means of micro-computed X-ray tomography (µCT) (EasyTom 160 from RX Solutions, Chavanod, France). The imaging process was accomplished at a voltage of 45 kV and a current of 160 mA using a micro-focused tube supplied with a tungsten filament. The source-to-detector distance (SDD) and the source-to-object distance (SOD) were updated in such a way to obtain a voxel size of around 2.3 µm. Volume reconstruction was achieved with the software Xact64 (RX Solutions) after adopting geometrical corrections and ring artefact attenuation. We performed 3D image analysis using the Avizo software 2022-2 (ThermoFisher, Waltham, MA, USA).

### 2.10. Compressive Strength

Ten samples of each group were kept in water for 24 h at 37 °C. After the immersion period, the specimens were subsequently analyzed through uniaxial compression testing to determine the maximum load before fracture. To that end, a universal electromechanical testing machine (Instron 3345, Norwood, MA, USA) was used with a 1 kN cell force, equipped with a displacement sensor. All the measurements were performed at a constant crosshead speed of 0.5 mm/min.

The compressive strength was calculated in megapascals (MPa) according to the following formula:

σc = 4P/πD2(1)

where P is the maximum recorded force during testing and D is the initial sample diameter.

### 2.11. Statistical Analysis

Statistical analysis was accomplished using SigmaPlot (release 11.2, Systat Software, Inc., San Jose, CA, USA). All the results were presented in means and standard deviations. Shapiro–Wilk testing was performed to check the normality. Analysis of variance on ranks (ANOVA) including a multiple comparison procedure (Tukey testing) was used to determine whether significant differences existed in the compressive strength values, flowability, pH, roughness, solubility and contact angle evaluations between the different sealers. In all the measurements, a statistical significance level α of 0.05 was adopted.

## 3. Results

### 3.1. pH Measurements

**Figure 2:**
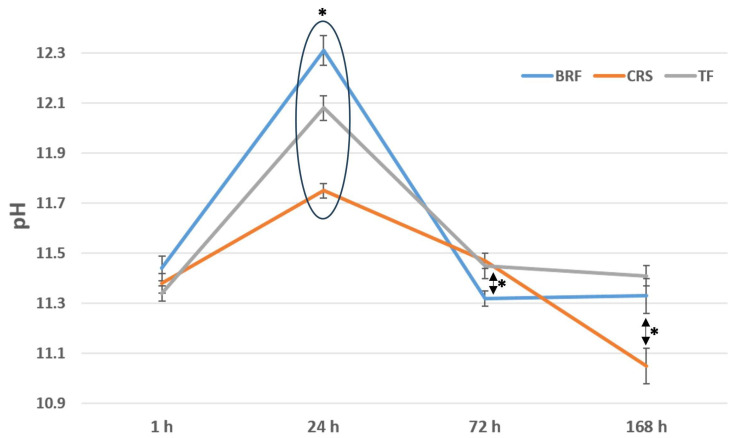
pH evolution with time (1, 24, 72 and 168 h) of distilled water at 37 °C in contact with BRF (BioRoot™ Flow), CRS (CeraSeal) and TF (TotalFill^®^). * *p* < 0.05.

All the sealers demonstrated an alkaline pH during the tested period (1–168 h). No significant difference was found between the sealers at 1 h, whilst BRF demonstrated a higher pH than TF and CRS at 24 h (*p* < 0.05). In addition, CRS showed a lower alkaline pH than the other sealers at 24 h (*p* < 0.05). No significant difference was found between CRS and TF (*p* > 0.05) at 72 h, whilst both sealers demonstrated higher pH values than BRF at 72 h (*p* < 0.05). After 7 days on incubation, no significant difference was found between TF and BRF (*p* > 0.05) and both demonstrated higher pH values than CRS (*p* < 0.05) (Figure 2).

### 3.2. Roughness and Wettability

**Figure 3:**
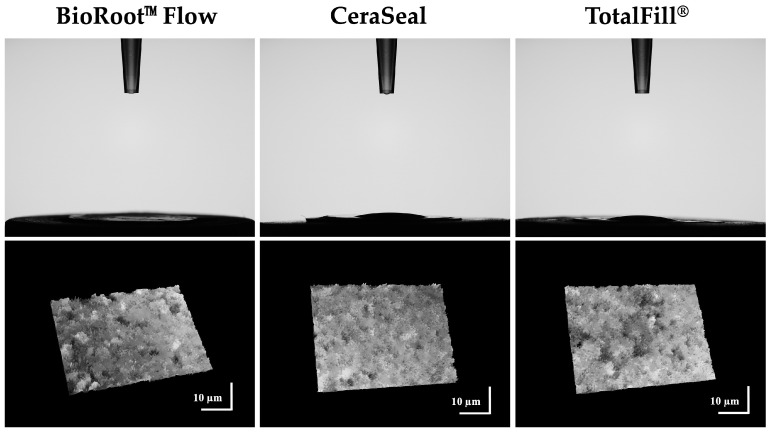
Water drop profiles on BRF (BioRoot™ Flow), CRS (CeraSeal) and TF (TotalFill^®^) surfaces after 10 s of water drop deposition. Digital micrographs of the different surfaces using KEYENCE 7000 VHX showing the roughness of each material.

After 10 s of deposition of the water drop, for BRF, we detected no water drop (0°) on the surface, whilst for CRS (16.8 ± 5.2°) and TF (12.5 ± 2.8°), we found hydrophilic surfaces with no significant difference between them (*p* > 0.05) (Figure 3).

A rougher surface was detected for TF (0.82 ± 0.21 µm) compared to BRF (0.58 ± 0.04 µm) (*p* < 0.05), whilst CRS (0.78 ± 0.15 µm) demonstrated no significant difference from TF or BR (*p* > 0.05).

### 3.3. Scanning Electron Microscopy

**Figure 4:**
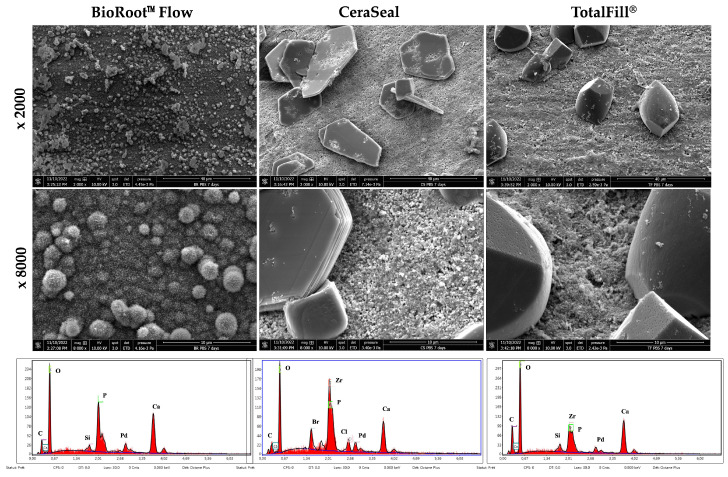
Scanning electron microscope images (2000× and 8000× magnification) demonstrating the mineral depositions on BRF (BioRoot™ Flow), CRS (CeraSeal) and TF (TotalFill^®^) surfaces after 7 days of immersion in PBS. EDX analysis demonstrates the chemical compositions of the different surfaces.

Differently shaped structures were observed on the different surfaces after 7 days of immersion in PBS (Figure 4). Urchin-like structures were observed on the BRF surface, hexagonal-shaped structures on the CRS surface and, finally, cubic forms on the TF surface. All the surfaces demonstrated different percentages of calcium, carbon, oxygen, silicium and phosphorus (Table 2).

### 3.4. Solubility and Flowability

The three sealers demonstrated solubility percentages that exceeded 3% after 24 h of immersion in water. TF (5.77 ± 0.08%) demonstrated higher solubility compared to BRF (4.52 ± 0.27%) and CRS (3.87 ± 0.11%) (*p* < 0.05). Moreover, CRS demonstrated a significantly lower solubility compared to TF and BRF (*p* < 0.05).

No significant difference was found for the flow properties between the three tested sealers (*p* < 0.05) (Table 3).

### 3.5. Porosity

**Figure 5:**
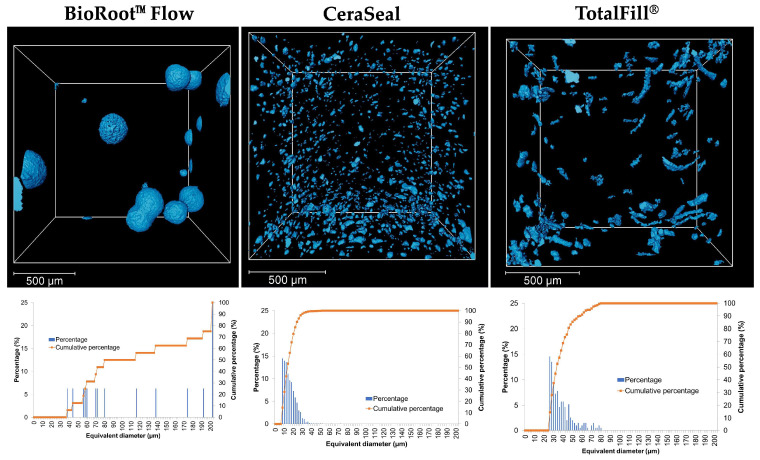
Volume rendering of segmented pores (blue color) with a scale bar of 500 µm, and equivalent pore diameter–frequency curves obtained by X-ray tomography analysis in BRF (BioRoot™ Flow), CRS (CeraSeal) and TF (TotalFill^®^).

A higher void volume fraction and void diameter were detected for BRF compared to CRS and TF (Table 3, Figure 5). As such, the calcium silicate-based sealers, CRS and TF, demonstrated similar porosities, which were different compared to the novel sealer, BRF. BRF demonstrated larger average porosity diameter (126.09 ± 13.88 µm) compared to TF (37.23 ± 3.05 µm) and CRS (14.83 ± 0.96 µm).

### 3.6. Compressive Strength

**Figure 6:**
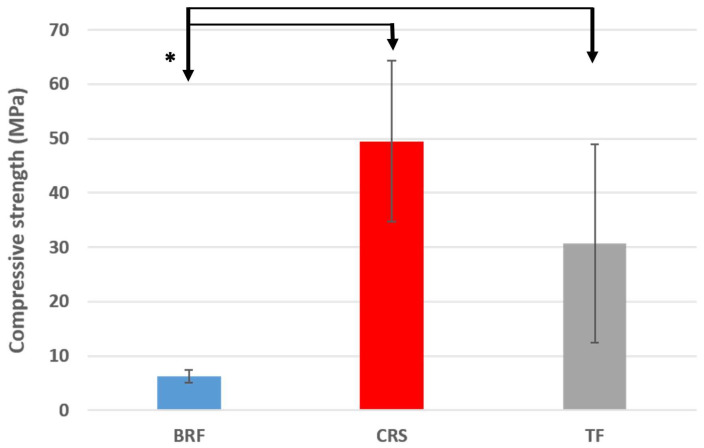
Means and standard deviations of compressive strength values for BRF (BioRoot™ Flow), CRS (CeraSeal) and TF (TotalFill^®^). * *p* < 0.05.

A lower significant compressive strength value was found for BRF (6.16 ± 1.13 MPa) compared to CRS (49.49 ± 13.26 MPa) and TF (30.71 ± 18.28 MPa) (*p* < 0.05). No significant difference was found between TF and CRS (*p* > 0.05) (Figure 6).

## 4. Discussion

The use of bioceramic materials in endodontic treatment is an essential for their biocompatibility and bioactivity [17]. Different formulations, preparations, application modes and chemical compositions of these materials are introduced in the dental market [18]. Previously, the researchers tried to find an optimal formulation of these materials that can avoid their negative sides. Several studies have noted the difficulty of retreatment of these materials, especially in the apical third [19,20]. Moreover, the solubility of these materials is known to be higher than those of other products used [21]. The novel product BioRoot™ Flow has been newly introduced in the dental market to replace the old powder–liquid version and thereby avoid the disadvantages of powder–liquid formulations. In this study, our aim was to evaluate the compressive strength and the physicochemical properties of the novel product and compare it to other bioceramic sealers.

The results of this in vitro study indicate that the null hypothesis should be rejected as there were significant differences between the three materials concerning their physicochemical and mechanical properties (*p* < 0.05).

The pH was evaluated at different time points for the three materials in contact with water at 37 °C. All three materials demonstrated an alkaline pH. There were significant differences between the different materials at different time points, but all their pH values from 1 h to 168 h were in the range between 10.5 and 12. This alkaline pH plays an important role in the antibacterial activity and healing process [22]. In accordance, several studies have demonstrated that water in contact with calcium silicate materials has an alkaline pH [3,12,22].

Different structures were observed on the material surfaces after 7 days of immersion in PBS. The use of PBS was to mimic the in vivo conditions of the fluids of dental tissues [23]. Urchin-like, hexagonal and cubic forms were observed on the BRF, CRS and TF surfaces, respectively. Several studies have reported that these structures could be observed on bioceramic materials [2,3,24]. The creation of these different structures could be associated with the different chemical compositions of the materials, as well as the pH, chemical elements and temperature of the storage environment [23,24,25,26,27].

The solubility of CRS was lower than those of the other both materials, whilst TF demonstrated higher solubility than BRF and CRS. These three sealers had solubilities of over 3% after 24 h of immersion in water at 37 °C. Accordingly, several studies have reported high solubility percentages for bioceramic materials [2,21]. Following the ISO standard 6876:2012 [16], an endodontic sealer should not present a solubility of more than 3% after 24 h of immersion in water. The bioactivity of these materials is related with the alkaline pH and the release of Ca^2+^ [2,3]; therefore, these materials should be soluble in order to release the particles needed for the bioactive effect. However, the higher solubility did not mean a higher pH or higher Ca^2+^ release. The bioactive effect and the solubility are not always correlated because the material can release other particles that do have any impact on the bioactive effect [28].

BRF demonstrated faster water adsorption compared to TF and CRS. After 10 s of deposition of 5 µL of water on the different sealer surfaces, the drop was totally adsorbed in the BRF group. TF and CRS demonstrated hydrophilic surfaces. A cutoff at 90 degrees has been accepted to define the hydrophobicity (>90°) and hydrophilicity (<90°) of materials’ surfaces [29]. The higher hydrophily of BRF could be related with the higher porosity and its chemical composition [30,31]. Several studies have reported a low or null contact angle for bioceramic products [12,30,31]. Moreover, the roughness of the surface may also affect the contact angle measurements [32]. CRS demonstrated no difference from TF and BRF in the surface roughness values, whilst TF demonstrated a rougher surface than BRF. A rougher surface and hydrophily play important roles in increasing protein adsorption, adhesion and cellular attachment [33,34,35]. In addition, not only is the value of roughness important for cell attachment but also the profile of this roughness is key for cell proliferation [36]. Finally, all three sealers have hydrophilic surfaces and good surface energy, which could influence the adhesion of these sealers to dentinal walls [31].

No significant difference was found between the three sealers concerning the flow properties. The importance of the flowability of an endodontic sealer or cement is directly related with the capacity of these materials to have a good filling ability and to penetrate the dentinal tubules and entomb the bacteria [37,38].

A lower compressive strength was detected for BRF compared to TF and CRS. This could be related to the higher porosity and average pore diameters that were detected in BRF samples compared to CRS and TF. The different chemical compositions of the three sealers as well as the pore sizes, morphology, distribution and connectivity could impact the compressive strength of calcium silicate materials [30]. The compressive strength of endodontic material has less importance in the root canal compared to the coronal part because the materials are not subjected to a high compressive strength [39]. Another study reported that the compressive strength of endodontic material plays an important role in reinforcing the prepared root canal [40]. One of the most important disadvantages of calcium silicate materials is their difficulty to be retreated and removed from a root canal [19,20]. This lower compressive strength of BRF could facilitate its removal from the root canal in the case of retreatment.

**Figure 7:**
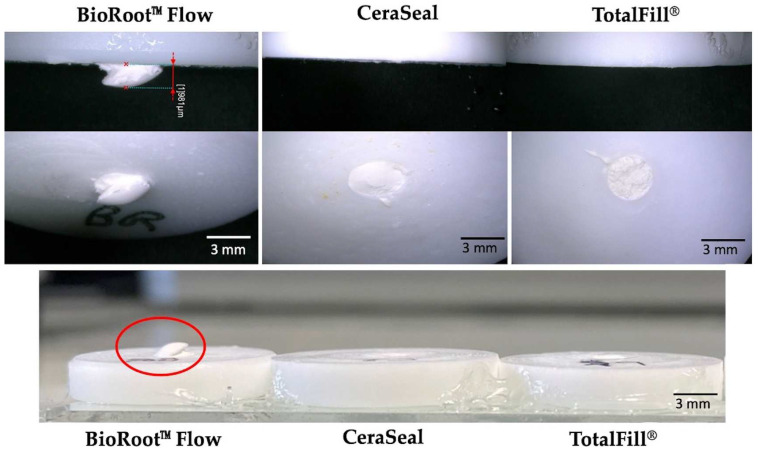
Digital images of the material expansion of BRF (BioRoot™ Flow), CRS (CeraSeal) and TF (TotalFill^®^) after 72 h of incubation at 37 °C. Red circle showed the expansion of BRF sealer.

During the preparation of the different samples using a Teflon mold, a clear expansion was detected for BRF samples after 72 h of setting in 37 °C and humidity (Figure 7). This expansion could be related to the fact that these materials setting in a moist environment results in the hydration of mineral oxide compounds to make various hydration products and the swelling of calcium silicate hydrate gel [41]. This process is responsible for the expansion of calcium silicate materials. Following the standard ISO 6876/2012 [16], this expansion should not exceed 0.1% and the shrinkage should not exceed 1.0% [42]. Some studies have demonstrated no shrinkage, whereas others have reported an expansion of up to 0.2%. Therefore, not all calcium silicate formulations could produce hygroscopic expansion [43]. Further studies, and quantitative testing of the expansion of the materials over time in the presence of humidity and temperature, should be performed.

One of this study’s limitations was its short storage period; a longer period should be employed to evaluate the effect of time on the solubility, crystallization and compressive strength of the novel product. In addition, X-ray diffraction (XRD) should be used in further studies in order to analyze the type of crystalline structure created on each surface. Moreover, in the present study, the pH values were analyzed over 168 h, and further work on a longer aging time should be performed to evaluate the associated pH changes. Moreover, further studies on the filling ability of these sealers should be performed as the quality of obturation is related to the quantity of voids and the apical sealing [1,2]. The biological side of these materials should also be evaluated, such as the antibacterial activity and cytotoxicity. Moreover, a further study could be performed to analyze the effect of these different sealers on the mechanical properties and fracture resistance of the teeth, as it was shown previously that bioceramic sealers could enhance the fracture resistance of teeth compared to others obturated with resin-based sealers [44].

Finally, several conditions may affect the success of root canal treatment and pain after treatment, such as the access cavity design [45], irrigation and cleaning protocol [46] and extrusion of debris after the shaping procedure [47,48]. In addition, the chemical composition of endodontic materials could play an important role in treatment success; thus, several research groups are searching for novel natural-origin materials that can be used to improve patients’ quality of life, ensure bioactivity and maintain high biocompatibility [49].

## 5. Conclusions

Within the limitations of the present in vitro study, the three calcium silicate-based sealers demonstrated good physicochemical properties. The three sealers have a high alkaline pH, which plays an important role in the antibacterial activity and healing process. All three sealers created shaped structure on their surfaces exhibiting hydrophilic properties. The novel sealer, BRF, demonstrated a lower significant compressive strength, which could facilitate the desobturation of this material during the endodontic retreatment process. To better evaluate the performance of endodontic sealers in laboratory tests, a simulation of the oral conditions may be required. Therefore, clinical trials using these calcium silicate-based sealers should be performed.

## Figures and Tables

**Table 1:** Manufacturers of the tested materials.

Sealer	Manufacturer	Lot	Manipulation
BioRoot™ Flow (BRF)	SEPTODONT Inc., Lancaster, PA, USA	B29518CAA	Premixed
CeraSeal (CRS)	Meta Biomed Europe GmbH, Mülheim an der Ruhr, Germany	CSL2209202	Premixed
TotalFill^®^ (TF)	FKG Dentaire SA, La Chaux-de-Fonds, France	21004SP	Premixed

**Table 2:** The mass percentages of the main elemental compounds of BRF (BioRoot™ Flow), CRS (CeraSeal) and TF (TotalFill^®^) surfaces in 95% humidity after 7 days in PBS at 37 °C.

Element	BRF	CRS	TF
O	32.61	32.08	35.99
Ca	30.53	22.40	27.17
P	12.21	1.03	2.56
Si	1.09	X	1.45
Zr	X	28.68	9.18

**Table 3:** Means and standard deviations of solubility percentages, flow properties and void characteristics of BRF (BioRoot™ Flow), CRS (CeraSeal) and TF (TotalFill^®^). Different superscript letters (a, b and c) indicate significant differences (*p* < 0.05).

Test	BRF	CRS	TF	Statistical Analysis
Solubility (%)	4.52 ± 0.27 ^a^	3.87 ± 0.11 ^b^	5.77 ± 0.08 ^c^	*p* < 0.05
Flow (cm)	2.65 ± 0.60	2.90 ± 0.08	2.46 ± 0.07	*p* > 0.05
Average void volume fraction (vol. %)	2.29 ± 0.73	0.42 ± 0.13	0.49 ± 0.17	
Average equivalent diameter (µm)	126.09 ± 13.88	14.83 ± 0.96	37.23 ± 3.05	

## Data Availability

The original contributions presented in the study are included in the article, further inquiries can be directed to the corresponding author.
